# Low Molecular Weight Adiponectin Increases the Mortality Risk in Very Old Patients

**DOI:** 10.14336/AD.2017.1117

**Published:** 2018-10-01

**Authors:** Stefano Rizza, Marina Cardellini, Alessio Farcomeni, Pasquale Morabito, Daniele Romanello, Giovanni Di Cola, Maria Paola Canale, Massimo Federici

**Affiliations:** ^1^Department of Systems Medicine, University of Rome Tor Vergata and; ^2^Center for Atherosclerosis, Policlinico Tor Vergata.; ^3^Department of Public Health and Infectious Diseases Sapienza, University of Rome, Italy

**Keywords:** adiponectin, isoforms, mortality, aging

## Abstract

Despite its beneficial role on insulin resistance and atherosclerosis, adiponectin has been frequently reported as an independent positive predictor of cardiovascular mortality. Very few information is available regarding adiponectin isoforms and mortality, in particular in advanced aging. Baseline serum levels of Total Adiponectin and its circulating isoforms (HMW-, MMW-, LMW-Adiponectin) were measured in 97 old patients (mean age: 79 years). Patients were followed up for all-cause mortality (study end-point) for an average of 76.4 ± 37.3 months. A positive association was observed for LMW-Ad and all-cause mortality (HR: 1.13, 95% CI: 1.05-1,22, p: 0.002). After multivariate adjustment for age, sex and a previous history of myocardial infarction, higher levels of LMW-Ad were significantly associated with all-cause mortality (HR: 1.11, 95% CI: 1.02-1.21; p*:* 0.017). Interestingly neither total adiponectin neither the other two circulating isoforms (MMW- and HMW-Ad) showed any significant association with the study end-point. Our data suggest that the association between high serum adiponectin levels and increased mortality rate in elderly is contingent to an unbalanced circulating levels of adiponectin isoforms. The present results support the hypothesis that high levels of Low Molecular Weight adiponectin are a biomarker for mortality risk in very old patients.

Adiponectin, the most abundant adipose-tissue hormone with pleiotropic actions in a plethora of tissues, is an independent positive predictor of mortality in the general population, especially in cardiovascular setting [1]. Nevertheless, adiponectin exerts many insulin-sensitizing, anti-inflammatory and endothelial protective effects [1]. Because of such paradox, it is logical to suspect that total adiponectin (Tot-Ad) level may not be a marker of cardiovascular mortality risk. Actually, adiponectin circulates in the blood as three discrete oligomeric complexes known as high-, medium- and low-molecular-weight adiponectin (HMW-, MMW- and LMW-Ad). While biological activities among these three isoforms are a matter of controversy, previous studies have indicated that HMW-, MMW- and LMW-Ad have different biological properties. However, the clinical data available on adiponectin isoforms are particularly restricted on its main isoform, the HMW-Ad, which is considered the active form [2].

Although HMW-Ad is known to have strong vasculoprotective and insulin-sensitizing properties, the majority of studies measured only Tot-Ad circulating levels; actually, only few studies have assessed the association of isomers levels and pathological conditions [3].

We previously reported that Tot-Ad level was higher in older adults with previous coronary heart disease (CHD) but, among circulating isoforms, only LMW-Ad was significantly associated with CHD in elderly patients [4]. Based on this report, we used a long follow-up to investigate a possible independent association between adiponectin circulating isoforms and total mortality in a group of very old patients.

## MATERIALS AND METHODS

This study was approved by the University of Tor Vergata Institutional-Review-Board and carried out in accordance with the principles of the Declaration of Helsinki as revised in 2000. Study patients were originally recruited for a clinical study which has been described in detail previously [4]. Follow-up information on outcome was collected yearly from 2002 to 2011 by a phone interview. The study outcome was all-cause mortality whose information was obtained and confirmed from patient’s primary care physicians, from death certificates or from review of web record (www.prescrizione.poslazio.it/sismed-prescription/assistito). Follow-up data are available for 97 subjects (mean age 83±5.4y) since, of 108 baseline individuals, 6 patients were excluded because their first-degree relatives disagreed to phone interview, 3 because changed phone addresses and 2 due to home region changing.

Smoking habits and history of hypertension, dyslipidemia and CHD as well as glucose-lowering treatment were also recorded at time of baseline examination. Data regarding medications were confirmed by review of medical records.

Serum high-sensitivity C-reactive protein (hsCRP) levels were measured using a nephelometric assay (Dade-Behring, Liederbach, Germany). Tumor necrosis factor alpha (TNF-α) levels were evaluated using enzyme-linked immunosorbent assay (ELISA) using commercially available kits (Bender MedSystem, Vienna, Austria). Adiponectin was measured using ELISA using a high-sensitivity commercial kit for Tot-Ad, HMW-Ad, MMW-Ad, and LMW-Ad isoforms (Alpco Diagnostics, Salem, NH). Estimated glomerular filtration rate (eGFR) was estimated according to creatinine-clearance using the Cockcroft-Gault formula.

### Statistical analysis

Patients’ characteristics were reported as mean and standard deviation (±SD) or median and Inter-Quartile-Range (IQR) for continuous variables as appropriate. Frequencies and percentages were used for categorical ones. Correlations between continuous variables were estimated using Spearman correlation. Time-to-event data were analyzed by means of log-rank test or univariate Cox-models for categorical or continuous variables, respectively. Significance of LMW-Ad was also assessed at multivariate Cox-modeling, after adjusting for possible relevant confounders. The maximum events to number of predictors ratio was fixed at fifteen, and the model was chosen by minimizing the Akaike Information Criterion (AIC) in a forward fashion. The following clinical mortality predictors were considered for final inclusion: age, sex, smoke, BMI, previous CHD, blood pressure, triglycerides, creatinin, fasting glucose, fasting insulin, HOMA IR, eGFR, HDL, LDL. Total-Ad, HMW-Ad, MMW-Ad, and LMW-Ad were forced in the model and considered separately for each of four multivariable Cox-regression models.

## RESULTS

Clinical characteristics of patients are summarized in Table 1. During follow-up (76.4 ± 37.3 months) 54 deaths occurred, corresponding to an overall annual incidence rate of 8.7%. Of those patients, 8 died for cardiovascular events (2 fatal-strokes and 6 fatal-myocardial-infarctions). When compared with the remaining participants, subjects who died during follow-up were older, more frequently men, had a higher prevalence of smoking habit and previous coronary artery disease but a similar rate of diabetes, eGFR and equivalent BMI, lipid profile, high-sensitivity CRP and TNF-α. Among Adiponectin isoforms, only LMW-Ad levels were significantly higher in subjects who died during follow up (2.3µg/ml vs 1.4µg/ml, respectively, p:0.024) (Table 1). Only in diabetic subjects LMW-Ad was positively associated with insulin resistance evaluated by HOMA IR (r:0.452, p:0.007) and in overall population LMW-Ad was significantly higher in male subjects (p:0.047) and in individuals with previous CHD (p:0.026) (data not showed).

Elevated levels of LMW-Ad were significantly associated with all-cause mortality (HR:1.13, 95%CI:1.05-1.22, p:0.002) whereas Total-, HMW- and MMW-Ad did not. To avoid possible confounding factors, we created four multivariate models, each for every adiponectin isoforms. After adjustment of covariates, including sex, age, and previous history of CHD, while neither Tot-Ad, nor HMW-Ad and MMW-Ad were associated with mortality (Table 2, Models 1,2 and 3), the hazard ratio for LMW-Ad was 1.11, again significant (95%CI:1.02-1.21; p:0.017) (Table 2, Model 4).

**Table 1:** Baseline clinical and laboratory characteristics of patients divided upon follow-up mortality. Data are displayed as n (%) or mean (±SD).

	All-cause deaths (n°54)	Survivors (n°43)	p
Age (years)	80.4±3.8	78.5±3.3	<0.05
Gender (male)	39(72.2)	21(48.8)	<0.056
BMI (kg/m^2^)	26.8±3.5	27.0±4.3	0.841
Systolic blood pressure (mmHg)	144.8±21.4	146.5±21.4	0.712
Diastolic blood pressure (mmHg)	75.6±12.4	77.6±10.0	0.427
Total cholesterol (mg/dl)	183.3±38.3	188.3±39.8	0.572
High-density-lipoprotein cholesterol (mg/dl)	48.7±13.2	54.3±14.7	0.085
Low-density-lipoprotein cholesterol (mg/dl)	118.3±32.8	116.5±34.7	0.813
Triglycerides (mg/dl)	123.8±61.6	110.3±37,3	0.220
Current or former smokers (yes)	32(59.2)	16(37.2)	<0.05
Diabetes (yes)	20(37.0)	11(25.5)	0.229
Stable CHD (yes)	24(44.4)	10(23.2)	<0.050
Tumor necrosis factor-α (pg/ml)	12.9±15.0	11.9±14.1	0.757
High sensitivity C-reactive protein (mg/l)	5.0±5.6	3.3±3.8	0.083
Estimated glomerul filtration rate (ml*sec-¹)	57.1±19.9	59.5±19.0	0.595
Total-Adiponectin (µg/ml)	4.7±4.2	4.6±4.1	0.896
HMW-Ad (µg/ml)	1.7±1.8	2.3±2.3	0.222
MMW-Ad (µg/ml)	1.0±1.0	1.1±1.3	0.831
LMW-Ad (µg/ml)	2.5±2.5	1.4±1.4	<0.05
Pharmacological Therapy			
Metformin* (%)	75	72	0.889
Sulphonylurea* (%)	25	36	0.504
Glitazone* (%)	10	27	0.210
Angiotensin-converting enzyme inhibitor or angiotensin II receptor antagonist (%)	77	83	0.826
Diuretic (%)	35	25	0.309
Calcium antagonist (%)	35	34	0.975
Beta-blocker (%)	50	42	0.424
Aspirin (%)	60	63	0.723
Statin (%)	57	55	0.942

CHD: coronary heart disease; HMW-Ad: High Molecular Weight-Adiponectin; MMW-Ad: Medium Molecular Weight-Adiponectin; LMW-Ad: Low Molecular Weight-Adiponectin

* Therapy referred to only diabetic subjects

**Table 2:** Multivariate analysis models for all-cause mortality with all Adiponectin isoforms.

	HR	CI (95%)	p
		**Model 1**	
Gender (male)	2.04	1.17-3.58	0.012
Age (year)	1.10	1.01-1.19	0.019
Stable CHD (yes)	1.45	0.80-2.64	0.220
Total-Adiponectin (µg/ml)	0.99	0.93-1.05	0.663
		**Model 2**	
Gender (male)	2.03	1.16-3.55	0.013
Age (year)	1.10	1.01-1.19	0.026
Stable CHD (yes)	1.45	0.80-2.65	0.220
HMW-Ad (µg/ml)	0.91	0.80-1.04	0.173
		**Model 3**	
Gender (male)	2.05	1.16-3.64	0.014
Age (year)	1.10	1.02-1.19	0.014
Stable CHD (yes)	1.35	0.73-2.49	0.337
MMW-Ad (µg/ml)	1.04	0.82-1.32	0.723
		**Model 4**	
Gender (male)	1.91	1.08-3.35	0.025
Age (year)	1.10	1.02-1.20	0.013
Stable CHD (yes)	1.22	0.67-2.21	0.51
LMW-Ad (µg/ml)	1.11	1.02-1.21	0.017

CHD: coronary heart disease; HMW-Ad: High Molecular

Weight-Adiponectin; MMW-Ad: Medium Molecular Weight-Adiponectin;

LMW-Ad: Low Molecular Weight-Adiponectin

## DISCUSSION

In this prospective study, after adjustment for cardiovascular mortality risk factors, we found a significant association between elevated levels of LMW-Ad and increased all-cause mortality in elderly patients.

The predictive capacity of LMW-Ad remained following adjustments for age, sex, and stable CHD. Of note, neither Tot-Ad, nor HMW-Ad and MMW-Ad were associated with all-cause mortality. Actually, subjects who died during the follow up had a large proportion of men than remaining participants. Generally, men have a shortened life expectancy. Thus, it is possible that this difference of gender may give a significant effect on mortality risk than that of LMW-adiponectin. Nevertheless, the predictive capacity of LMW-Ad remained significant even when survival analysis was performed only in male subjects (LogRank 4.44, p:0.035).

To the best of our knowledge, this is the first report concerning the relathionship between multimeric adiponectin forms and mortality in aged people. Previous studies linking adiponectin levels and mortality in older adults have taken into account only Tot-Ad concentration, missing to investigate the distribution of adiponectin isoforms.

The mechanism behind the positive association of Tot-Ad and mortality is not fully understood and at a first glance seems contradictory as adiponectin has anti-inflammatory and anti-atherogenic properties. This particular relationship has been described as a paradox [1].

Nevertheless, in specific cell types, adiponectin exerts some deleterious effects on processes involved in atherogenesis and in oncogenesis, including stimulatory effect on secretion of pro-inflammatory cytokines and angiogenesis [5-7]. Furthermore, some reports concluded that increased plasma adiponectin level is an independent risk for dementia and Alzheimer disease in human cohorts [8].

A significant increase of adiponectin is reported in chronic inflammatory related disease such as rheumatoid arthritis, systemic lupus erythematosus, inflammatory bowel diseases, allergy and asthma [9].

However, since our results come from elderly subjects it is intriguing to speculate that, in a state of healthy aging without critical vascular, proliferative or infective diseases, adiponectin levels may be suppressed or lead anti-inflammatory signals, while in frailer patient adiponectin may be driven upward as an attempt of a natural defense and determine the activation of detrimental signals beside the well-known anti-inflammatory pathways. Whether Adiponectin Receptor 1 and 2 play different roles in this context is unknown, although recently it has been shown that the two receptors extert opposite effects on cancer progression [10-11]. The observed increase in LMW-Ad in our and other studies might be dependent from an impaired multimerization process, that is observed in chronic disorders such as diabetes and during aging. Consistently, a recent cross-sectional study in subjects with RA showed that LMW-Ad is associated with disease activity [12].

Our study has some limitations. First, the study enrolled a limited number of subjects although included a follow up for mortality having adiponectin multimers data available at baseline. Second, it is possible that subjects with low LMW-Ad levels were already dead at the beginning of the study, affecting the main result. Consequently, it is feasible that LMW-Ad plays an indirect effect on total mortality, that may be related to the age of the subjects. Third, low HMW-Ad was higher in survivors but not significantly associated to mortality and we cannot exclude that this is dependent by the number of events or subjects in our study.

In previous report [4] we observed that LMW-Ad was significantly higher in CAD subjects without renal and heart failure. Nevertheless, although it is possible that LMW-Ad might be protecting especially to the heart and vasculature, due to the limited numbers of CV deaths, this work does not support a specific cardiovascular role for LMW-Ad. Finally, the study was from a single European district, but it is known that adiponectin levels differ according to ethnicity.

In conclusion, our pilot observation in elderly subjects suggests that measuring LMW-Ad levels may help to improve the risk of all cause mortality, a finding that might deserve replication in larger cohorts and different ethnicities.
